# Potential therapeutic effects of peroxisome proliferator-activated receptors on corneal diseases

**DOI:** 10.3389/ebm.2024.10142

**Published:** 2024-06-27

**Authors:** Bing Jie Chow, Isabelle Xin Yu Lee, Chang Liu, Yu-Chi Liu

**Affiliations:** ^1^ Barts and the London School of Medicine and Dentistry, Queen Mary University of London, London, United Kingdom; ^2^ Tissue Engineering and Cell Therapy Group, Singapore Eye Research Institute, Singapore, Singapore; ^3^ Department of Cornea and External Eye Disease, Singapore National Eye Centre, Singapore, Singapore; ^4^ Ophthalmology and Visual Sciences Academic Clinical Program, Duke-National University of Singapore (NUS) Medical School, Singapore, Singapore

**Keywords:** cornea, proliferator-activated receptors, neovascularisation, wound healing, inflammation, fibrosis, nerve regeneration

## Abstract

The cornea is an avascular tissue in the eye that has multiple functions in the eye to maintain clear vision which can significantly impair one’s vision when subjected to damage. Peroxisome proliferator-activated receptors (PPARs), a family of nuclear receptor proteins comprising three different peroxisome proliferator-activated receptor (PPAR) isoforms, namely, PPAR alpha (α), PPAR gamma (γ), and PPAR delta (δ), have emerged as potential therapeutic targets for treating corneal diseases. In this review, we summarised the current literature on the therapeutic effects of PPAR agents on corneal diseases. We discussed the role of PPARs in the modulation of corneal wound healing, suppression of corneal inflammation, neovascularisation, fibrosis, stimulation of corneal nerve regeneration, and amelioration of dry eye by inhibiting oxidative stress within the cornea. We also discussed the underlying mechanisms of these therapeutic effects. Future clinical trials are warranted to further attest to the clinical therapeutic efficacy.

## Impact statement

The cornea constitutes a vital element of the ocular structure which exerts a profound impact on vision when compromised. The application of peroxisome proliferator-activated receptor (PPAR) agents has been widely documented particularly in the treatment of metabolic conditions such as hyperlipidemia and diabetes. Yet, their therapeutic potential in the context of corneal diseases remains not well-understood. This review article summarises the reported therapeutic effects of PPAR agents in the management of corneal inflammation, neovascularisation, wound healing, fibrosis, nerve regeneration, and dry eye. With further clinical validation, PPAR agents may serve as a new avenue in the treatment of a variety of corneal diseases.

## The cornea

The cornea is an avascular tissue that sits at the anterior-most surface of the eye. It comprises both cellular and acellular components; the former includes epithelial cells, keratocytes, and endothelial cells, and the latter includes mainly collagen and glycosaminoglycans.

Functionally, the cornea fulfills several crucial roles, including protecting ocular integrity, preserving optical clarity, and providing refractive power to the eye. The cornea provides a protective barrier against the environment, facilitated by the intercellular junctions within its epithelium and the continuous regeneration of the corneal epithelial cells [1, 2]. Consequently, damage to the cornea through trauma renders it compromised, which in turn triggers a corneal wound healing response consisting of inflammatory and fibrotic reactions. Excessive corneal wound healing responses lead to corneal scarring and opacification, profoundly impacting vision. The World Health Organization stated that cornea opacity is a priority eye disease and is one of the main causes of low vision and visual impairment, affecting 146 million people worldwide with an increase of 1.5–2.0 million new cases every year [3, 4]. This underscores the importance of corneal wound healing process, emphasizing a need to develop more efficacious interventions to accelerate this restorative process.

The cornea is also the most densely innervated tissue in our body, richly supplied by sensory and autonomic nerve fibres [5]. Corneal nerves sustain corneal health and homeostasis by facilitating tear secretion and providing trophic support to epithelial and stromal cells [6, 7]. In conditions such as diabetic keratopathy or corneal surgery, corneal nerve degeneration manifests, causing decreased corneal sensitivity and increased vulnerability to corneal ulceration [8, 9]. Therefore, interventions targeting corneal nerve regeneration constitute a pivotal approach to enhancing cornea health.

Researchers have previously explored potential therapeutic agents aimed at these fundamental aspects of corneal health. In this review, we explore a promising therapeutic avenue, namely, the peroxisome proliferator-activated receptor family, on corneal diseases.

## The expression of PPARs in eyes

The PPARs are a group of transcription factors belonging to the nuclear hormone receptor family [10]. The PPAR family comprises three isotypes: PPAR alpha (α), PPAR gamma (γ), and PPAR delta (δ), each exhibiting distinct tissue expression patterns and involved in the regulation of diverse biological functions [11]. At the molecular level, while the PPAR isoforms exhibit a sequence identity of 60%–70% within their ligand-binding domains (LBDs), significant variations in the overall pocket size of the three-dimensional structures of these LBDs exist, leading to distinctive binding affinities of each PPAR isoform with specific compounds [12]. The discovery of PPAR’s mechanism as a distinct transcription factor capable of targeted activation by peroxisome proliferators for potential therapeutic application, by Issemann and Green in 1990, paved the groundwork for further research into PPAR [13]. Since then, prior research has firmly established the role of PPARs as modulators of adipocyte differentiation, glucose and lipid metabolism, and inflammation [11, 13, 14]. Within the eye, all 3 PPAR isoforms exhibit distinct localisation patterns within ocular structures [15]. Specifically, PPARα and PPARγ are found in the cornea, conjunctiva, retina, meibomian, and lacrimal glands, whereas PPARδ is expressed in the cornea, retina, and lacrimal glands, all demonstrating varied levels of expression across various tissue types [15, 16]. Beyond the eye, PPARα is localised in the kidneys, liver, muscle, and heart; PPARδ displays ubiquitously across numerous organs and tissues, while PPARγ is found in adipocytes and small intestines [17, 18]. Clinically, they are routinely used as therapeutic agents such as fenofibrate, a PPARα agonist, for hyperlipidaemia, as well as pioglitazone, a PPARγ agonist for diabetes [19, 20].

All 3 PPAR isoforms have been reported to exhibit expression within the retina, each with differing functions. PPARα has been reported to possess protective and anti-inflammatory effects in the retina across several studies [21–24]. A study reported the PPARα’s anti-apoptotic properties in retinal ischaemia [21], whilst another demonstrated anti-oxidative and anti-angiogenic effects of PPARα in age-related macular degeneration [22]. In a similar vein, PPARα has illustrated their anti-inflammatory role in treating diabetic retinopathy and experimental autoimmune uveoretinitis [23, 24]. PPARγ has been reported to exhibit neuroprotective effects on retinal ganglion cells, preventing retinal dysfunction following optic nerve crush [25]. The existing therapeutic options to address corneal pathological processes remains limited. Within corneal inflammation, topical corticosteroid or non-steroidal anti-inflammatory drugs (NSAIDs) serve as the main agents for treatment. However, both anti-inflammatory treatment options are accompanied by significant adverse side-effects, including increased intraocular pressure, cataract formation due to corticosteroid use [26], and NSAID-induced corneal melting [27]. The current treatment options of steroids or mitomycin C for corneal fibrosis also present with limitations with particular concerns pertaining to long-term drug safety profile [28]. Lastly, recombinant nerve growth factor (NGF) emerges as the singular drug approved by the Food and Drug Administration (FDA) to treat neurotrophic keratopathy. However, this treatment option remains costly and dictates the need for frequent topical administration [29]. Given that inflammation and angiogenesis are closely associated with corneal scar tissue formation, the various therapeutic effects of PPAR agents, positions them as promising therapeutic candidates for corneal diseases. Additionally, several PPAR agents such as fenofibrate and rosiglitazone are existing drugs widely used in the treatment of metabolic diseases, offering the advantages of drug repurposing in contrast to *de novo* drug discovery. This includes reduced developmental time and costs, alongside well-established pharmacokinetic considerations [30]. In this review, we focus on the therapeutic potential and underlying work of action of the different PPAR isoforms on corneal diseases, including corneal wound healing, corneal inflammation, fibrosis, neovascularisation, corneal nerve regeneration, and dry eye disease.

### PPARs on corneal wound healing

Corneal wound healing is a complex process driven by local molecular factors and endogenous soluble factors. It is a widely accepted notion that transforming growth factor-beta (TGF-β) and tumour necrosis factor alpha (TNF-α) play pivotal roles in regulating cellular responses during wound healing [31, 32]. Previous studies examined the 3 PPAR isoforms in relation to corneal wound healing, uncovering mechanisms that accelerate the process.

PPARα’s involvement in corneal wound healing has been recently elucidated through its role in regulating corneal cell metabolism [33]. *In vitro* studies have revealed that mitochondrial oxidative phosphorylation is a primary source of adenosine triphosphate (ATP) production for human corneal epithelial cells. Significantly reduced mitochondria metabolism and subsequently, impaired corneal healing process in PPARα knockout mice, compared to wild-type mice, were found, suggesting the role of PPARα as a key regulator of mitochondrial metabolism and corneal wound healing. In addition, PPARα expression was downregulated in diabetic human corneas compared to non-diabetic groups, reinforcing the role of PPARα in mitochondria metabolism and corneal healing in light of well-documented delayed wound healing observed in diabetic corneas. Administration of fenofibrate, a PPARα agonist, ameliorated mitochondrial dysfunction and enhanced corneal wound healing in diabetic mice and humans, further underscoring the role of PPARα in corneal wound healing [33].

Regarding the role of PPARγ in corneal wound healing, *in-vitro* epithelial cell proliferation was significantly accelerated following adenoviral gene transfer of PPARγ [34]. Additionally, PPARγ effectively preserved the corneal epithelial basement membrane in the alkali-burned corneas subjected to adenoviral gene transfer. Expression of matrix metalloproteinase-2 (MMP-2) and TGF-β within the corneal epithelium was significantly suppressed by PPARγ gene transfer using real-time RT-PCR [34]. Given the TGF-β’s inhibitory role in epithelial cell growth and MMP’s role in extracellular matrix degradation, this indicates PPARγ’s participation in corneal wound healing.

PPARδ has also been shown to promote corneal healing via facilitating proliferative capacity in rat alkali burn models [35]. This was evidenced by significantly increased Ki67-positive cells and Ki67 mRNA expression following four topical administration of 0.05% GW50516 solution, a PPARδ agonist [35]. Compensatory elevation of PPARδ expression during corneal wound healing in both animal and human corneal models was reported, and topical administration of PPARδ agonist further inhibited corneal epithelial cell death, thereby facilitating corneal wound healing [36].

### PPARs on corneal inflammation

PPARα’s involvement in corneal inflammation has been explored by assessing the anti-inflammatory effects in rat corneal chemical injury models [37, 38]. Significant suppression of inflammatory cell infiltration was observed in the cornea following alkali burn after the instillation of 0.05% fenofibrate twice daily for 14 days, in comparison to the vehicle group [38]. Western blotting also demonstrated a significantly reduced expression of nuclear factor-kappa B (NF-κB), a key transcription factor in inflammation, in the PPARα group versus the vehicle group [38].

Similarly, another study investigating the anti-inflammatory effects of fenofibrate following rat corneal alkali injury demonstrated a reduction in corneal inflammatory processes [37]. This was evidenced by significantly reduced mRNA expression of proinflammatory cytokines and chemokines such as interleukin-1 (IL-1), IL-6, IL-8 and monocyte chemoattractant protein-1 (MCP-1) [37]. Following corneal chemical injury, staining with a PPARα antibody highlighted the primary localisation of PPARα-positive cells within the regenerating epithelial basement cell, indicating the vital role of PPARα in inflammatory responses [37].

PPARγ was also explored for its potential corneal anti-inflammatory effects [39]. Similar to the effects observed with PPARα, topical application of PPARγ agonist, specifically pioglitazone hydrochloride, significantly decreased neutrophilic and macrophage infiltration after alkali-burn injury in rats, whilst significantly increasing anti-inflammatory M2 macrophages. Further real-time reverse transcription polymerase chain reaction (RT-PCR) analysis revealed the suppression of IL-1, IL-6, IL-8, TGF-β1, and MCP-1 in corneas [39]. Similarly, in another study using an alkali injury model, a significant reduction in TNF-α mRNA expression and upregulation of M2 macrophage polarisation upon PPARγ agonist treatment were observed, enhancing the role of PPARγ in the context of corneal inflammation [40].

Topical application of a synthetic PPARδ-specific agonist, GW5015116, twice daily for 7 days, also significantly inhibited neutrophil and macrophage infiltration in rat corneal alkali burn models. Significantly lower expression levels of NF-Κb and inflammatory cytokines were also observed in the PPARδ group compared to the vehicle group on the real-time RT-PCR analysis [41].

The anti-inflammatory effects of the PPAR agonists within the cornea have been postulated to be orchestrated through several pathways. Western blotting analyses indicate that the interference of the PPAR isoforms with activity of key proinflammatory transcription factors such as NF-kB pathway, which may underlie the significantly decreased immune cell infiltration observed in the cornea across these studies. This is in line with previous studies which has shown that the attenuation of NF-kB activity by PPARα agents may be achieved through the maintenance of a negative regulator, nuclear expression of the kappa light polypeptide gene enhancer in the B cell inhibitor, alpha (I_k_B-α), which plays a role in inhibiting NF-kB activation [42]. Additionally, PPAR agonists may exert immunomodulatory effects in corneal inflammation by enhancing monocyte differentiation to M2 macrophages. In the context of macrophage-driven inflammation, M1 macrophages are distinguished as tissue injury-type macrophages which serve as potent effector cells that kill microorganisms and produce pro-inflammatory cytokines such as IL-6 and TNF-α, inducing inflammation [43]. Conversely, M2 macrophages function to dampen inflammation through the production of anti-inflammatory factors, promoting tissue remodelling and repair [44]. The decrease in pro-inflammatory cytokines like MCP-1 may additionally contribute to the reduced immune cell infiltration, given its crucial role in promoting immune cell infiltration [45]. Thus, the observed reduction in pro-inflammatory cytokine expression in these studies may be ascribed to the immunomodulatory effects of PPAR agonists during corneal inflammation.

### Suppression of corneal fibrosis of PPARs

Corneal fibrosis arises from abnormalities in corneal wound healing, characterised by excessive production of aberrant extracellular matrix (ECM) proteins and corneal crystalline enzymes by myofibroblasts. Whilst corneal fibrogenesis acts to restore corneal integrity following injury, excessive wound remodeling causes corneal scars, resulting in visual impairment or blindness [46]. In corneal fibrogenesis, TGF-β1 is a key cytokine responsible for promoting keratocyte differentiation into active myofibroblasts [47, 48]. The profibrotic properties of TGF-β1 are achieved through multiple intracellular signaling pathways such as Smad, p38 mitogen-activated protein kinase (MAPK), and extracellular signal-regulated kinase (Erk) [49, 50]. Given the established anti-fibrotic properties of PPARγ in the lungs and kidneys [51, 52], corneal studies have explored the possible role of PPARγ in alleviating corneal scarring [53–56].

Instillations of pioglitazone, a PPARγ agonist, demonstrated a significant inhibitory effect on corneal fibroblast migration, conducted on cultured corneal fibroblasts using scrape-wound assays [53]. It also led to a significant reduction in corneal lattice contraction, as illustrated by significantly greater lattice diameters in corneal fibroblasts seeded in free-floating collagen gels. Significant decreases in matrix metalloproteinase-2 (MMP-2) and MMP-9 secretion, alongside significantly reduced collagen I and fibronectin protein synthesis, demonstrated in western blotting, were also observed.

Moreover, PPARγ downregulated the expression of TGF-β1-induced connective tissue growth factor (CTGF), which is a major autocrine growth factor enhancing TGF-β1’s pro-fibrogenic role in myofibroblast differentiation [54]. This highlights PPARγ’s anti-fibrotic effect on corneal fibroblasts by inhibiting crucial components in developing corneal scarring. PPARγ is involved in two distinct profibrotic signaling pathways, specifically in the p38 MAPK and Smad signalling pathways. Within the p38 MAPK signalling pathway, PPARγ ligands, including troglitazone, rosiglitazone, and 15d-PGJ2, significantly reduced the levels of phosphorylated p38 MAPK in a dose-dependent manner [55]. Addition of PPARγ ligands down-regulated β-catenin expression—a component of p38 MAPK signalling by blocking the TGF-β1-induced p38 MAPK phosphorylation. The involvement of β-catenin acts as a downstream mediator of p38 MAPK signalling, significantly enhancing α-SMA production in western blot analysis [56].

PPARγ’s anti-fibrotic action in the TGF-β1-induced Smad signalling has been demonstrated through the application of lobeglitazone, a PPARγ agonist, in a combination of type I collagen and corneal fibroblast isolated from the human stroma. Notably, western blot analysis of Smad2/3 and P-Smad2, key proteins in the Smad signalling pathway, indicated a significant inhibition in Smad signalling and myofibroblast differentiation [57].

## The application of PPARs for corneal nerve regeneration

Degeneration of corneal nerves can occur in conditions such as diabetic keratopathy, which is a common microvascular complication of diabetes. This arises from the accumulation of advanced glycation end products and the generation of reactive oxygen species (ROS) [58], triggered by prolonged hyperglycaemia, which reduces microvascular supply to Schwann cells and neurons through increased oxidative stress and inflammation impacting the capillaries [59]. As hyperlipidemia is a known risk factor for diabetic neuropathy [8], PPARα has been explored for its potential therapeutic effects in diabetic corneal neuropathy.

PPARα-knockout mice presented with significant decrease in corneal nerve fiber density (CNFD), as well as significantly decreased corneal sensitivity, compared to the wild-type mice [60], suggesting PPARα’s supportive role in maintaining corneal nerve health. The protective role of PPARα agonist in diabetic corneal neuropathy further revealed that fenofibrate had a significantly positive effect in ameliorating corneal nerve degeneration in diabetic rat models. A restoration of PPARα expression in corneal epithelium and significantly increased CNFD were observed following treatment with chow containing 0.014% fenofibrate, a PPARα agonist, for 4 months [60]. Our group further demonstrated that topical fenofibrate eye drops enhanced the CNFD, corneal nerve fiber length, and ocular surface integrity, as well as suppressed the corneal neuroinflammation, in diabetic keratopathy [61].

**FIGURE 1:**
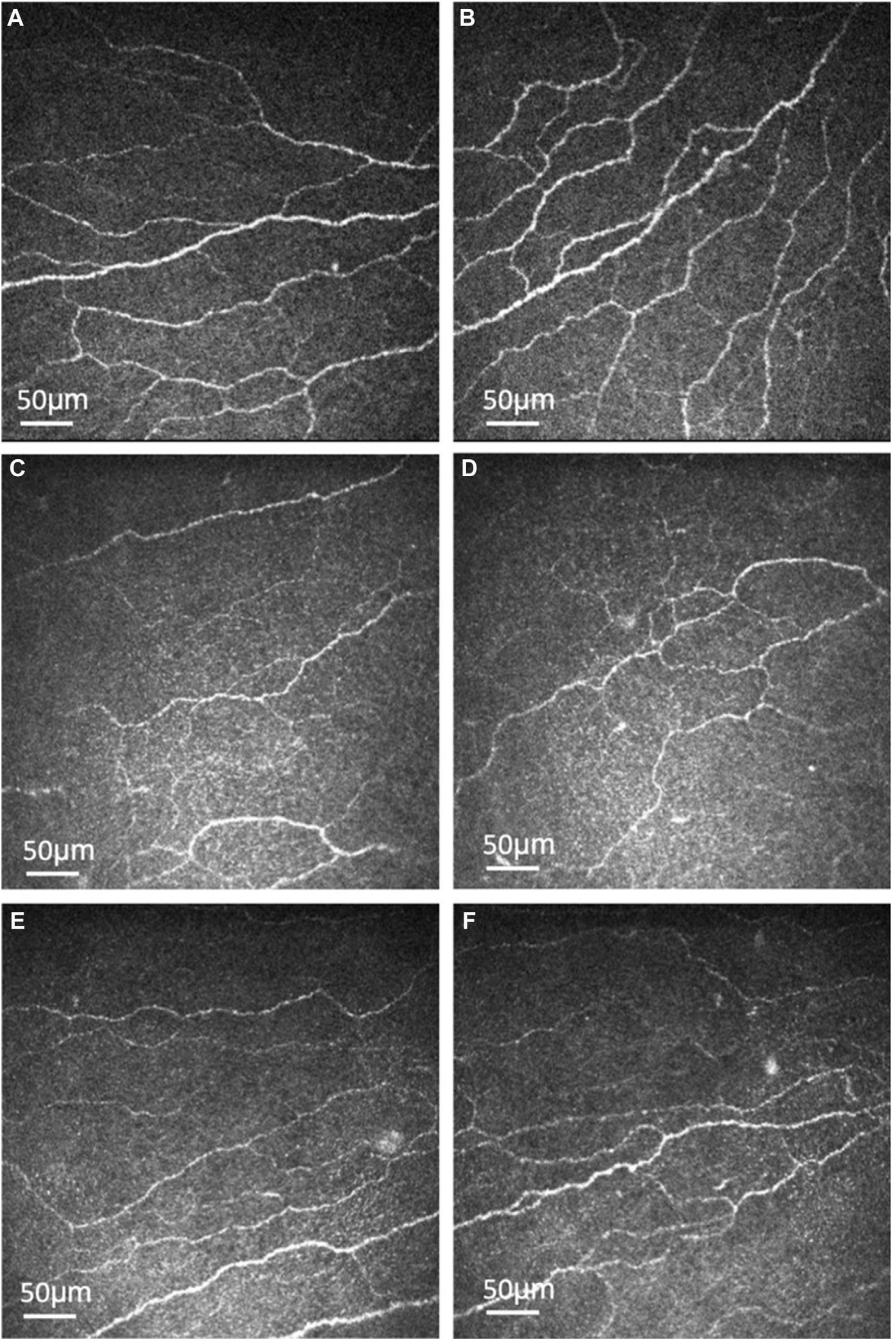
Representative corneal subbasal nerve images of healthy controls and type 2 diabetic patients before and after oral fenofibrate treatment. Corneal subbasal nerve of healthy controls **(A, B)**. Corneal subbasal nerve before **(C**, **D)** and after **(E**, **F)** oral fenofibrate treatment, demonstrating an increase in nerve fiber density (9.25 *± 4.24* vs*.* 18.99 ± 8.49 n/mm^2^) and corneal nerve fiber width (0.0215 ± 0.0002 vs. 0.0224 ± 0.0005 µm^2^/mm^2^) after treatment.

More recently, our group published a clinical trial in which 30 patients with type 2 DM were treated with oral fenofibrate for 1 month. On *in vivo* confocal microscopy evaluation, there was significant stimulation of corneal nerve regeneration and a reduction in nerve oedema after oral fenofibrate treatment, as evidenced by significant improvement in CNFD and corneal nerve fiber width, respectively (Figure 1). There was also a significant improvement in the corneal epithelial cell morphology in terms of cell circularity. More importantly, fenofibrate significantly improved patients’ neuropathic ocular surface status by increasing tear breakup time along with a reduction of corneal and conjunctival punctate keratopathy. Amelioration of ocular surface neuroinflammatory status was also found, evidenced by a significant increase in tear substance P level. On the quantitative proteomic analysis, fenofibrate significantly upregulated and modulated the neurotrophin, MAPK signaling pathways and linoleic acid (LA) metabolism, which may account for the neurotrophic effects of fenofibrate clinically [62; 63]. In addition, the expression of proteins involved in the regulation of nervous system function such as beta-galactoside alpha-2,6-sialytransferase 1 (ST6GAL1), tetratricopeptide repeat protein 9A (TTC9), ras-related protein (RAB5A) and suppressor of mothers against decapentaplegic homolog 1 (SMAD1) were significantly increased post-treatment [63]. On the pathway analysis, we identified the following potential underlying therapeutic mechanisms in corneal nerve regeneration: 1) Upregulation of neuronal pathways 2) lipid modulation, 3) anti-inflammation and 4) anti-coagulation.

**FIGURE 2:**
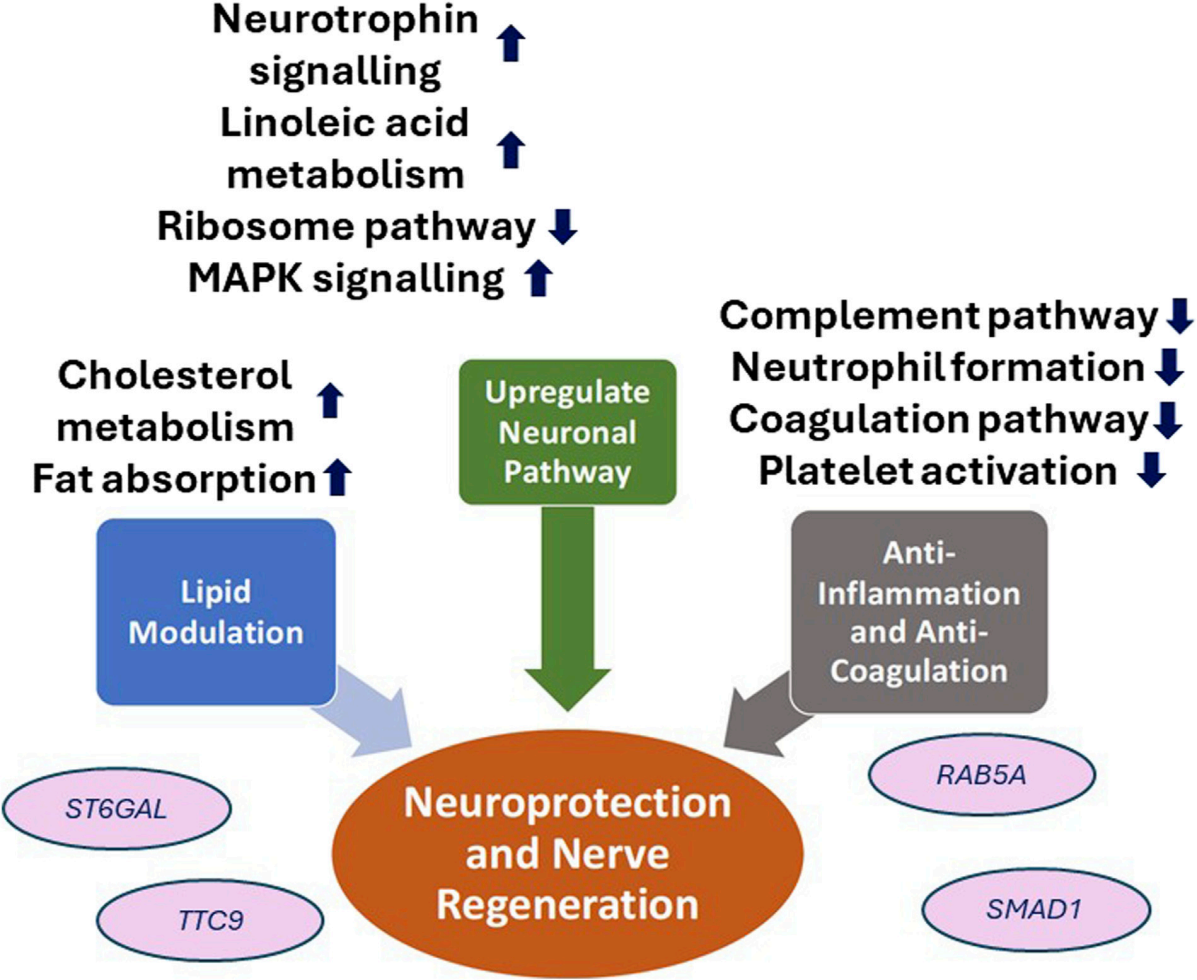
Schematic diagram of the neuroprotective effects of PPARα agent, fenofibrate, in diabetic corneal neuropathy. ST6GAL1, Beta-galactoside alpha-2,6-sialytransferase 1; TTC9, Tetratricopeptide repeat protein 9A; RAB5A, ras-related protein; SMAD1, Suppressor of mothers against decapentaplegic homolog 1.

Fenofibrate demonstrated an upregulation of the neurotrophin, MAPK signaling pathway and linoleic acid metabolism which are crucial key players in neuroprotection. Neurotrophins are a class of growth factors that regulate neuronal development, survival, death and plasticity [6]. Within the cornea, neutrophins facilitates corneal nerve branching, maintenance of corneal nerve density, and promoting nerve regeneration [7]. Activation of MAPK has been demonstrated to mediate neurite outgrowth-promoting effects *in vitro* [64]. In addition, the metabolism of LA is highly important given its role of gamma LA production, an vital component of neuronal membrane phospholipid, as well as playing a role to preserve nervous blood flow to coordinate nerve regeneration [65]. Suppression of the ribosome family expression by fenofibrate may also account for its neuroprotective effects [63], particularly as axonal ribosomes are associated as a marker for diseased axons in neurogenerative conditions (Figure 2) [66].

The therapeutic effect of fenofibrate in corneal regeneration and peripheral nerve improvements may also be attributed to its well-established effect as an anti-hyperlipidaemic agent. Fenofibrate’s antihyperlipidaemic effect is expressed through the stimulation of lipoprotein lipase activation, which promotes the synthesis of high-density lipoprotein (HDL) cholesterol and fatty acid oxidation pathway whilst conversely facilitating a rapid degradation of low-density lipoprotein (LDL) and triglycerides within tissues [67]. Given that hypertriglyceridemia, hyperlipidemia and decreased HDLc serve as significant risk factors for diabetic peripheral neuropathy, the lipid-modulating effects of fenofibrate may partly account for its neuroprotective impact in both peripheral neuropathy and corneal nerve regeneration. Other studies have also postulated that the peripheral neuroprotective role of PPARα agents is achieved through PPARα activation in satellite glial cells of dorsal root ganglia to stimulate axon regeneration and the activation of the PPAR-α-AMPK-PGC-1α pathway to ameliorate neuronal and endothelial damage [68–70]. This is also reinforced in the Fenofibrate Intervention and Event Lowering in Diabetes (FIELD) study which reported that fenofibrate significantly reduced microvascular complications such as diabetic neuropathy alongside a 37% risk reduction of amputation in T2DM patients [71].

The anti-inflammatory effect of fenofibrate through the suppression of NF-κB expression, as explored earlier, also plays a neuroprotective role through the reduction of neuroinflammation whilst promoting neurodevelopmental processes such as neurogenesis, neuritogenesis and axoneogenesis [40, 72–74]. Fenofibrate has also been reported to play a role in anti-coagulation by inhibiting complement and coagulation cascades alongside platelet activation pathways [63]. We contend that this results in knock-on effects on fenofibrate’s neuroprotective effects given the significant association between low platelet time and platelecrit levels with decreased nerve conduction function and prevalence of neuropathy in T2DM patients [75].

## PPARs on dry eye disease

Dry eye diseases have multifaceted origins and are characterised by a loss of homeostasis of the tear film [76]. These conditions are accompanied by ocular symptoms, including tear film instability, hyperosmolarity, ocular surface inflammation and damage, and neurosensory abnormalities [77, 78]. Dry eye conditions have been associated with oxidative stress [79], and diabetes given that the generation of oxidative stress is a core pathological mechanism of diabetes [80]. The pathogenic processes associated with oxidative stress encompass a disruption in the equilibrium between the oxidative and antioxidant systems, culminating in hindered neutralisation of oxygen free radicals [81]. The crucial role of PPARγ expression in the lacrimal and meibomian glands and dry eye has been explored in mouse models [16]. Qualitative PCR revealed that rats with dry eyes had significantly decreased PPARγ expression compared to healthy rats [16]. PPARγ agents have been shown to directly influence the transcription of antioxidants through the activation of PPAR response elements located in their respective promoter regions, such as catalases, superoxide dismutases 1 and 2 [82]. As such, they have been employed in the inhibition of the occurrence of oxidative stress [83].

Rosiglitazone, a PPARγ agonist, has been evaluated in the context of diabetes-related and hyperlipidemia-related dry eye in mouse models [82, 84]. Daily rosiglitazone administration effectively reduced ROS accumulation in the lacrimal glands of diabetes-related dry eye rat models in 4 weeks [82]. This effect was observed through a significant decrease in ROS fluorescein intensity in the rosiglitazone-treated group, distinguishing itself from both the non-treatment and vehicle groups of diabetes-related dry eye rat models [82].

Further real-time RT-PCR analysis revealed significantly increased mRNA expression levels of antioxidant enzymes glutathione peroxidase 3 (GPx3) and heme oxygenase-1 (HO-1) in the lacrimal gland of the rosiglitazone-treated group [82]. Tear production was also significantly increased following rosiglitazone administration. Apart from its beneficial effects in stimulating tear production in the lacrimal glands, it alleviated ocular surface damage as evidenced by significant improvements in corneal fluorescein staining score compared to the non-treatment group [82]. Additionally, rosiglitazone led to significantly decreased ROS levels within the cornea, determined through comparisons of ROS fluorescein intensity [82]. In a separate study, rosiglitazone effectively reduced pro-inflammatory cytokines and inflammatory cell infiltration within the meibomian glands of hyperlipidaemic rat models, yielding favourable effects in alleviating meibomian gland dysfunction and evaporative dry eye disease [84]. These findings highlight the potential therapeutic role of PPARγ in the management of dry eye disease.

Fenofibrate, a PPARα agonist, has been reported to suppress the formation of ocular surface squamous metaplasia, a pathological process of dry eye disease. In a rat model study where tear film instability was induced by topical benzalkonium chloride (BAC), topical fenofibrate demonstrated a reduction in abnormal corneal epidermal differentiation [85]. This reduction was evidenced by decreased expression of K10 keratin, an epidermal keratinocyte-specific intermediate filament, within the corneal epithelium of the fenofibrate-treated group compared to the non-treatment/vehicle groups through immunostaining [85]. Additionally, objective markers evaluating tear film instability, including corneal fluorescein sodium staining scoring was significantly improved in the fenofibrate-treated [85]. As such, fenofibrate has displayed its potential effectiveness in reducing the inflammatory response and offering a treatment option for use as a preventive agent in patients with high risks of dry eye [85].

## PPARs on corneal neovascularisation

Corneal angiogenesis typically occurs in wound healing and tissue repair. The pathogenesis of corneal neovascularisation stems from an interplay of the disequilibrium between proangiogenic and anti-angiogenic factors [86]. Keratocytes have been suggested to be integral in corneal neovascularisation formation given their expression of vascular endothelial growth factors (VEGFs) and MMP-13. These factors are identified to degrade type 1 collagen in the cornea, thereby creating an environment conducive to corneal neovascularisation [87]. While the mainstay initial treatment for neovascular ophthalmopathy involves suppressing endothelial cell growth through anti-VEGF agents, it is accompanied by limitations including suboptimal treatment response, short-effect duration, and side effects [88]. With established PPARα expression in vascular endothelium and prior findings indicating fenofibrate’s protective effects against retinal vasculopathy by inhibiting vascular endothelium function, combined with PPARγ’s potent molecular inhibition of angiogenesis [89, 90], assessing PPAR agents as a therapeutic target holds promise for the management of corneal neovascularisation.

The expression of PPARα has demonstrated a notably inverse correlation with corneal neovascularisation formation and upregulation of VEGFr3 and MMP13 in alkali-burned corneas of wild-type and PPAR-knockout mice [91]. Additionally, in PPARα knockout mice, significantly higher VEGFr3 and MMP13 levels were exhibited versus wild-type mice following corneal alkali burns. Wild-type mice that were subjected to corneal alkali burn were divided into two groups and administered with 200 μM topical fenofibrate or vehicle solution daily for 5 days to investigate fenofibrate’s impact on corneal neovascularisation. Corneal revascularisation was significantly reduced in the fenofibrate-treated group upon clinical slit-lamp examination compared to the vehicle group [91]. In another study, fenofibrate effectively reduced the expression of VEGF mRNA, as well as angiopoietin-1 (Ang-1) and Ang-2, which are proangiogenic factors, in post-alkali burn corneas. These findings suggest that PPARα may play an inhibitory role in the context of neovascularisation [38].

Sarayba et al. investigated the influence of PPARγ on corneal neovascularisation formation in rats with three experimental groups: implanted pellets containing both pioglitazone and VEGF, pellets containing VEGF alone, and controls. Quantitative image analysis based on digital ocular photographs demonstrated a significant reduction in the mean density of corneal neovascularisation formation in the corneas of the VEGF/PPARγ group in comparison to the VEGF group at day 7 after implantation, although there was no statistically significant difference in the mean corneal neovascularisation area between these two groups. This still illustrates, however, the potential anti-angiogenic effect of PPARγ agonist, in mitigating corneal neovascularisation [92].

**FIGURE 3:**
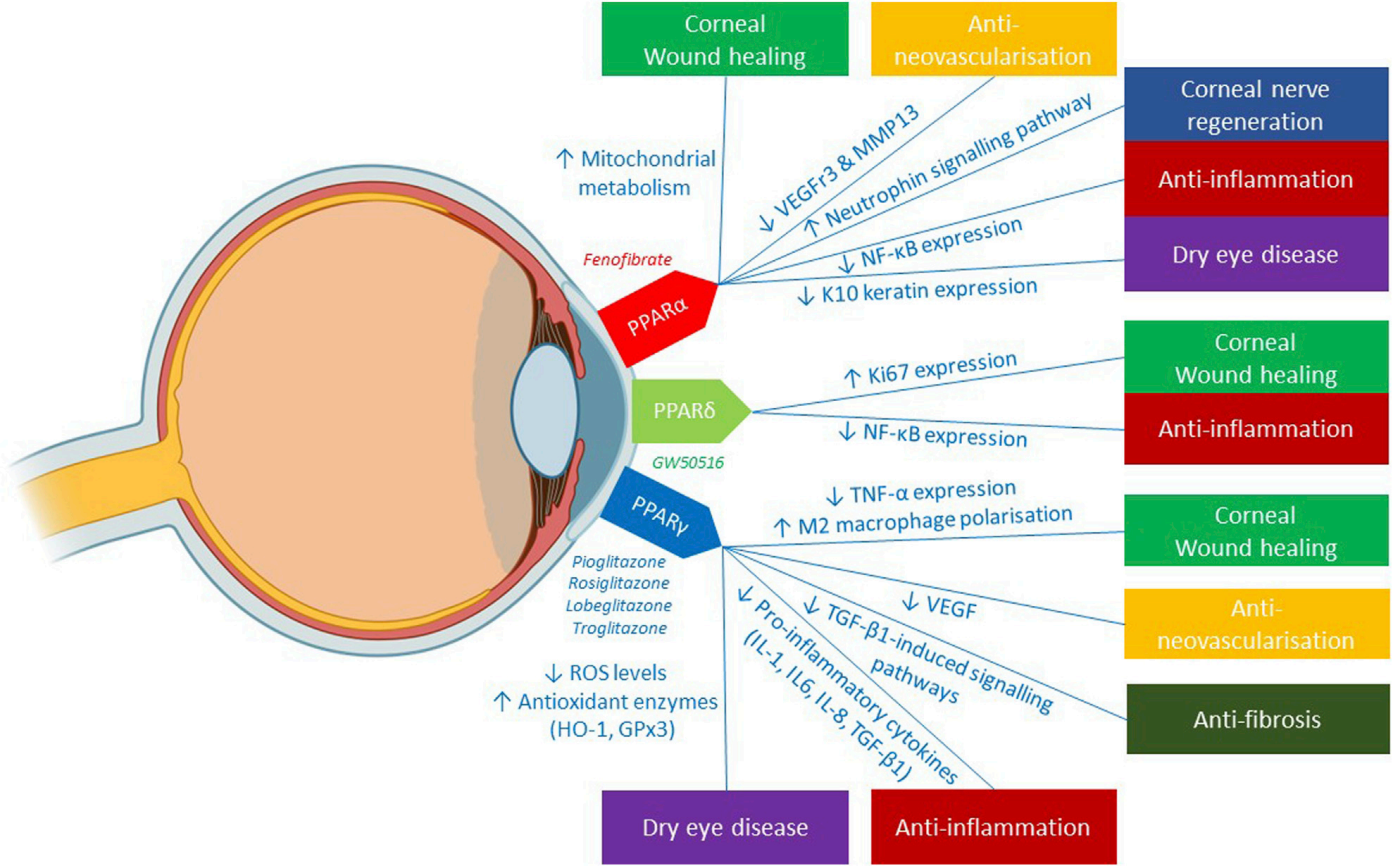
Illustration of the clinical applications of 3 PPAR isoforms in corneal diseases and its underlying mechanisms reported in the literature. Figure was created with BioRender.com.

Figure 3 summarizes the potentials and mechanisms of the 3 PPAR isoforms (PPARα, PPARγ, PPARδ) in mitigating the pathogenesis of various corneal diseases. Each of the 3 PPAR classes exhibited therapeutic effects in promoting corneal wound healing via distinct mechanisms: 1) PPARα enhanced the main energy source of corneal epithelial cells, specifically mitochondrial metabolism 2) PPARδ augmented corneal proliferative capacity, as illustrated by increased expression of Ki67, a marker of cell proliferation. 3) PPARγ contributed to the inhibition of MMP-2 and TGF-β, enzymes that impede corneal wound healing. The anti-inflammatory capabilities of PPAR agents have been evidenced by the modulation of key inflammatory transcription factor, NF-κB via PPARα and PPARδ and the pro-inflammatory cytokine expression by PPARγ. In the realm of anti-neovascularisation, the suppression of pro-angiogenic factors like VEGFr3 and MMP13 by PPARα, as well as VEGF by PPARγ confers anti-angiogenic benefits in managing corneal neovascularisation. The therapeutic effects of PPAR agents in dry eye diseases has been delineated through two main mechanisms: firstly, the anti-oxidative function of PPARγ in diminishing corneal ROS levels, thereby mitigating ocular surface damage; and secondly, the role of PPARα in impeding aberrant corneal epidermal differentiation and consequent formation of ocular surface squamous metaplasia—a late sequelae of dry eye disease. The corneal neurotrophic effects of PPARα agent, fenofibrate, has also been highlighted through the enhanced activation of neuronal pathways involving MAPK, neurotrophin and LA metabolism. Additionally, fenofibrate’s active role in anti-inflammation, anticoagulation, and lipid modulation contributes to secondary neurotrophic effects, creating an optimal environment for corneal nerve regeneration.

## Clinical safety of PPAR agents

At present, the research of PPAR agents on ocular diseases remains limited, with most studies focusing on *in-vitro* and animal experiments, highlighting a paucity of data on the safety of topical PPAR agents in corneal diseases, However, the clinical application of systemic PPAR agents, notably the PPARα agonists belonging to the fibrates class such as fenofibrate and PPARγ agonists from the thiazolidinedione (TZD) class such as rosiglitazone, are well-validated in the treatment of dyslipidaemia and diabetes, respectively, with an established safety profile [19, 20].

Systemic administration of the fibrate drug class such as fenofibrate exhibits a relatively favourable side-effect profile, with minor associated adverse side-effects spanning from gastrointestinal discomfort, musculoskeletal symptoms, to headaches [15]. Rare instances of rhabdomyolysis has also been described in concurrent use of statin-fibrate therapy, and highlights an increased risk in patients with hypothyroidism, renal disease and diabetes mellitus [93]. Adverse effects of the TZD class includes adverse cardiovascular effects, particularly fluid retention, leading to congestive heart failure and peripheral tissue oedema [94]. Whilst a potential association between TZD use and the development of diabetic macular oedema in diabetic patients has been postulated [95], this remains a topic of debate with subsequent studies reporting no association between the TZD use and diabetic macular oedema [96, 97]. Conversely, there is limited available data regarding the safety of PPARδ agonists. Of note, the early clinical trials involving the PPARδ agonists, GW501516, were halted due to concerns over accelerated carcinogenicity observed in animal models [98].

## Future directions

While the concept of utilising PPAR agents in corneal diseases has gained momentum in recent years, additional efforts are needed to further elucidate the role of the specific PPAR isoforms. Firstly, there remains a need for a deeper understanding of the specific PPAR isoforms involved in specific corneal diseases. As illustrated in this review, different isoforms play distinct roles and further delineation of their functions within the cornea could open avenues for additional therapeutic interventions. Understanding the crosstalk between PPAR and other signalling pathways implicated in corneal diseases could uncover synergistic or antagonistic effects, providing a comprehensive picture of PPAR’s role in maintaining corneal homeostasis.

Currently, the studies discussed in this article largely employ the utilisation of animal models, showcasing great promise and broadening our knowledge base on the underlying mechanisms of PPAR agents in treating corneal diseases while serving as a platform to test these novel PPAR therapeutic modalities. Further directions on this aspect may include using *in-vitro* three-dimensional human corneal models and human corneal cell culture models, reducing reliance on animal models, and offering the advantage of enhanced physiological resemblance to *in-vivo* studies. Additionally, conducting clinical trials assessing the efficacy and the long-term safety profile of PPAR agonists in treating specific corneal diseases in a real-world setting is imperative. Rigorous evaluation of the PPAR agents in diverse patient populations will validate their therapeutic potential and guide optimal dosing regimens.

Beyond the future endeavours aimed at establishing PPAR agents’ efficacy in the landscape of corneal disease management, there remains a considerable scope to develop novel drug application techniques to bolster the efficacy of current PPAR agents for improved clinical outcomes. The conventional delivery method for the treatment of corneal diseases involves the application of therapeutic agents via topical eye drops. However, it presents with its own limitations, including rapid precorneal drug loss and inability to sustain therapeutic drug concentrations over extended periods [99]. Through nanomedicine which utilises the use of nano-particles as carriers to treat diseases, nano-based ocular delivery may offer a more optimal drug delivery profile, specifically targeting desired corneal cells to intercept pathological pathways [100]. The application of lipid nanoparticles for lipophilic agents like fenofibrate presents a promising avenue, particularly in light of its poor bioavailability due to limited penetration of corneal epithelium [101]. Within the realm of PPAR agents, the utilisation of nanomedicine as a carrier for PPARγ agent has been tested for the treatment of chronic liver disease and have shown to reduce liver fibrosis and inflammation [102]. As such, these studies provide insight into the feasibility of nanomedicine as an innovative delivery platform for PPAR formulations in future. By amalgamating these diverse research trajectories, the field can anticipate a more nuanced understanding and application of PPARs in the therapeutic landscape of corneal diseases.

## Conclusion

This article has reviewed current studies detailing the therapeutic effects of PPAR agents in various corneal diseases. Many studies have validated the potential therapeutic effects of PPAR agents in addressing aspects of corneal pathology, including corneal wound healing, neovascularisation, inflammation, fibrosis, nerve regeneration, and dry eye disease. Future studies may involve more in-depth examination of the specific PPAR isoforms in corneal diseases and progress towards the integration of clinical trials, to further attest the beneficial roles of PPAR agents in corneal diseases.
